# Hair Cortisol/DHEA-S Ratios in Healthcare Workers and Their Patients During the COVID-19 Pandemic: A Case Study

**DOI:** 10.3390/life14121582

**Published:** 2024-12-01

**Authors:** Isabella Pividori, Tanja Peric, Antonella Comin, Alessio Cotticelli, Mirco Corazzin, Alberto Prandi, Massimo Domenico Mascolo

**Affiliations:** 1 Department of Agricultural, Food, Environmental and Animal Sciences, University of Udine, 33100 Udine, Italy; isabella.pividori@uniud.it (I.P.); tanja.peric@uniud.it (T.P.); antonella.comin@uniud.it (A.C.); alberto.prandi@uniud.it (A.P.); 2 Department of Veterinary Medicine and Animal Production, Federico II University, 80137 Naples, Italy; alessio.cotticelli@unina.it; 3 Department of Neurology, Hospital of San Giorgio, Via Gemelli 10, 33170 Pordenone, Italy; neuro@massimomascolo.it

**Keywords:** COVID-19, hair cortisol/dehydroepiandrosterone sulfate, hair, healthcare workers, neurological degenerative diseases

## Abstract

Background: Unlike psychological distress, which has been extensively studied during the COVID-19 pandemic, the impact of the pandemic on stress hormones has been overlooked. The aim of this study is to examine the hair cortisol/dehydroepiandrosterone sulfate (DHEA-S) ratios as markers of HPA axis dysregulation in healthcare workers and their patients. Methods: A total of 200 healthcare workers and 161 “patients” patients with special healthcare needs due to chronic illness or motor disabilities were included in this study. The hormone concentrations were measured using a radioimmunoassay. Results: Our results show that the patients had significantly higher cortisol/DHEA-S ratios than the workers. A high cortisol/DHEA-S ratio in the patients reflects higher cortisol concentrations (*p* < 0.001) and lower DHEA-S (*p* < 0.05) concentrations compared to those of the healthcare workers, suggesting that they may be exposed to a greater degree of stress and a decrease in their ability to cope with their disease. The cut-off value of the hair cortisol/DHEA-S ratio in our study for detecting people with needs that require special consideration and attention was 1.46 (*p* ≤ 0.01). Conclusions: Assessing the hair cortisol/DHEA-S ratios in both healthcare workers and the patients allowed us to identify a non-homeostatic condition that could lead to disease and to understand psychophysical well-being during the COVID-19 pandemic. They also play a crucial role in preventive and personalized medicine.

## 1. Introduction

The COVID-19 pandemic has affected the healthcare system worldwide, especially healthcare professionals. Psychological distress during the COVID-19 pandemic has been extensively studied in both health workers and patients, while its impact on stress hormones has been mostly overlooked. An objective measure, such as a stress hormones assessment, does not exclude subjective measures, but can have complementary utility in delineating a multidimensional framework. Three months after the onset of the COVID-19 pandemic, Cyr et al. (2021) [1] found an increase in hair cortisol concentrations and an association between burnout status and changes in cortisol in healthcare workers [1]. The effects of this critical situation, which was characterized by increased pressure due to daily contact with patients, high time demands, a risk of infection, and inadequate protection, exposed them to a risk of developing mental health symptoms.

Despite the efforts of these workers, COVID-19 has even increased stress, anxiety, and depression in their patients suffering from degenerative neurological diseases. This population appears to be one of the most vulnerable, as it is affected by incurable diseases that require long-term care. Hu et al. (2021) [2] observed that this increase in stress had an impact on mental and psychological health and caused accelerated neurodegeneration in patients suffering from neurological degenerative diseases.

Long-term stress can lead to hypothalamic–pituitary–adrenal (HPA) axis dysregulation with abnormal concentrations of the HPA axis hormones. Cortisol has neurotoxic effects under conditions of prolonged or repeated stress exposure, but is essential for the proper functioning of the body and brain, as it regulates numerous basal processes [3]. This hormone has profound effects on metabolism, which include causing central obesity, hepatic steatosis, insulin resistance, and may hence contribute to acquiring metabolic syndrome (MetS) [4].

Many studies have concerned the determination of cortisol, while dehydroepiandrosterone sulfate (DHEA-S) has only recently encountered some interest. DHEA-S, like DHEA, exhibits a general neurostimulation effect that appears to counterbalance many of the negative effects of cortisol and can be a protective factor against an excessive response to cortisol in stressful situations [5,6]. DHEA-S also regulates adipose tissue metabolism by modulating the risk of diabetes and MetS [4].

Considering the synchronized synthesis of these two hormones and their opposing effects [7,8,9], the cortisol/DHEA-S ratio may be more informative than the absolute concentrations [10]. The disruption of the dynamic balance of these two hormones, especially a higher cortisol/DHEA-S ratio, might signal susceptibility to the dysregulation of HPA axis activity [11], have an impact on metabolic, cognitive, and psychological function, and increase the risk for mental and physical health problems more vulnerable to disease (e.g., neurodegenerative, cardiovascular, immune dysregulations, and metabolic problems) [6,12,13]. The cortisol/DHEA-S ratio has been found to be positively associated with MetS, and high cortisol concentrations were associated with an increased risk of MetS, while high DHEA-S concentrations appeared to be protective [14] to MetS.

The concentrations of cortisol and DHEA-S can be assayed in various biological matrices, each reflecting a specific timeframe of the HPA axis activation. The measurement of these hormones in blood serum, saliva, and urine provides information on their concentrations at a single point in time, while the use of hair allows for retrospective determination through a single strand of hair [15]. The hair allows for evaluating the long-term exposure in an objective way, and the sampling is not invasive or painful. The samples can be easily stored at room temperature for an extended period [16], and they have low susceptibility to typical confounding factors, such as circadian and ultradian rhythmicity [17].

The aim of this study was to examine the hair cortisol/DHEA-S ratio in healthcare workers and their patients during the COVID-19 pandemic. The stress generated by the work–family–pandemic conflict is a daily challenge for healthcare professionals who must constantly guarantee an ad hoc approach for degenerative neurological patients. These are patients who have a debilitating pathology and a rehabilitation strategy whose effectiveness could be compromised by problems related to the consequences of the historical period.

## 2. Materials and Methods

### 2.1. Ethical Approval

The study was carried out in accordance with the Declaration of Helsinki and approved by the Ethics Committee of the University of Udine (protocol number 0000842). Furthermore, the study was conducted in compliance with Directive (EU) 2016/679 on the collection and use of data. The participants did not receive any kind of compensation for participating in the study, and all of them gave informed consent.

### 2.2. Study Population

In this study, healthcare workers were recruited along with patients hospitalized in a rehabilitation integrative center three months after the COVID-19 pandemic was declared a health emergency in Italy (March 2020).

The study consisted of 361 hair samples. In total, 161 were taken from patients (74 ± 13 years; mean ± SD; 113 women, 48 men) recruited from “Ospizio Marino di Grado” (GO, Italy) a rehabilitation integrative center. All patients hospitalized in this structure and included in the study were affected by neurological degenerative diseases.

In total, 200 hair samples were taken from healthy healthcare workers (53 ± 18 years; mean ± SD, 137 women, 63 men) who were recruited from the same hospital and members of the staff of health workers.

No person included in the study tested positive for COVID-19. Smokers, pregnant women, people with adrenocortical dysfunctions, and those characterized by the use of substances, oral contraceptives, hair dyes, anti-dandruff shampoos, frequent washing, or other hair treatments were excluded from the study.

### 2.3. Hair Sampling

The hair samples were collected from the patients and healthcare workers starting three months after the beginning of the COVID-19 pandemic. For the patients, the hair samples were collected seven days after entering the rehabilitation facility.

The hair was sampled (125 mg) non-invasively and painlessly, on the *vertex posterior* region of the head using commercially available vacuum hair clippers. Steroid concentrations were determined from the 1 cm segment of hair closest to the scalp.

The hair matrix has a fairly predictable growth rate of approximately 1 cm/month [18], and, therefore, the 1 cm segment closest to the scalp approximates the last month’s hormonal production [16]. A lag time of approximately two weeks should be considered for the deposition of steroid hormones into hair because of its initial deposition beneath the skin [19]. In addition, hormones captured inside the hair are stable and can be stored for years [20,21]. Each sample was stored in a paper envelope at room temperature and protected from UV rays until it was processed.

### 2.4. Reagents, Consumables, and Instruments

Isopropanol (2-Propanol; ≥99.8% (GC)) and methanol (≥99.8% (GC)) were purchased from Sigma-Aldrich (Merck KGaA, Darmstadt, Germany), while the phosphate-buffered saline (PBS, 0.05 M, pH 7.5 (RIA buffer)) and the 0.15 mM sodium acetate buffer (pH 9) were prepared in our lab, with all of the reagents purchased from the same supplier as the solvents above. Analyses were performed in 96-well microtiter plates (OptiPlate 96-well, white, high binding) purchased from Perkin-Elmer Life Science (Boston, MA, USA). The goat anti-rabbit γ-globulin serum and the rabbit anti-cortisol serum (CORT-3-CMO-BSA) were obtained from Analytical Antibodies (Bologna, Italy), while the rabbit anti-DHEA-S serum (SDHEA-7ßCM-BSA) was purchased from Bertin Bioreagent (Montigny le Bretonneux, France). The standards used were as follows: Hydrocortisone, ≥98% (HPLC), CAS Registry Number 50-23-7, Molecular Weight 362.46, and Dehydroisoandrosterone 3-sulfate sodium salt dihydrate, ≥93% (TLC), CAS Registry Number 78590-17-7, Molecular Weight 426.50, both purchased from Sigma-Aldrich (Merck KGaA, Darmstadt, Germany). The tracers were Hydrocortisone (Cortisol, [1,2,6,7-3H(N)]-), concentration 1.0 mCi/mL, molecular weight 362.4, and Dehydroepiandrosterone Sulfate, Sodium Salt, [1,2,6,7-3H(N)]-, concentration 1.0 mCi/mL, molecular weight 390.5, both purchased from Perkin-Elmer Life Science (Boston, MA, USA). The liquid scintillation cocktail MicroScintTM-20, designed for microplate formats, was purchased from Perkin-Elmer Life Science (Boston, MA, USA). Plates were counted on the β-counter Top-Count (Perkin-Elmer Life Science, Boston, MA, USA).

### 2.5. Sample Preparation and Extraction

Forty-five milligrams of the first segment (length 1 cm) of hair were weighted, and each hair strand was washed twice using H_2_O for 3′, and then, in agreement with Davenport et al. (2006) [22], with isopropanol for 3′. These stages allow us to minimize the risk of extracting cortisol from outside the hair and ensure the removal of sweat and sebaceous secretions from the external surface of the hair.

Steroids was extracted by incubating each specimen for 16 h in methanol at 37 °C. Next, the liquid in the glass vial was evaporated to dryness at 37 °C under an airstream suction hood. The remaining residue was dissolved in 1.2 mL of phosphate-buffered saline (PBS), 0.05 M, pH 7.5 (RIA buffer).

### 2.6. Cortisol and DHEA-S Radioimmunoassay Method

The concentrations of cortisol and DHEA-S were measured using solid-phase microtiter radioimmunoassay (RIA). In brief, a 96-well microtiter plate was coated with goat anti-rabbit γ-globulin serum diluted 1:1000 in 0.15 mM sodium acetate buffer (pH 9) and incubated overnight at 4 °C. The plate was then washed twice with RIA buffer (pH 7.5) and incubated overnight at 4 °C with 200 μL of the antibody cortisol serum diluted at ratios of 1:20,000, and 200 μL of the anti-DHEA-S serum diluted at 1:800. The cross-reactivities of the anti-cortisol antibody with other steroids were as follows: cortisol, 100%; cortisone 4.3%; corticosterone 2.8%; 11-deoxycorticosterone 0.7%; 17-hydroxyprogesterone 0.6%; dexamethasone 0.1%; progesterone, 17-hydroxypregnenolone, DHEAS, androsterone sulfate, and pregnenolone < 0.01%. The cross-reactivities of DHEA-S antibody with other steroids are as follows: DHEA-S 100%, 4-Androsten-3,17-dione (4-Androstenedione) 0.2%, 4-Androsten-17-ol-3-one (Testosterone) ≤ 0.01%, 5-Androsten-3-ol-17-one (Dehydroepiandrosterone, DHEA) ≤ 0.01%, and 5-Androstan-3-ol-17-one (Androsterone) ≤ 0.01%. After washing the plate with RIA buffer, the standards (100–4000 pg/mL), the quality-control extract, the test extracts, and the tracer (hydrocortisone {cortisol [1,2,6,7-3H (N)]; DHEA-S [1,2,6,7-3H (N)]) were added, and the plate was incubated overnight at 4 °C. The bound hormones were separated from the free hormones by decanting and washing the wells in the RIA buffer. After the addition of 200 μL of scintillation cocktail, the plate was counted on a β-counter.

The intra- and inter-assay coefficients of variation were, for cortisol, 6.4% and 13.9%, respectively. For DHEA-S, the intra- and inter-assay coefficients of variation were 7.0% and 10.3%, respectively. The sensitivities of the assays were 24.6 and 15.8 pg/mL for cortisol and DHEA-S, respectively.

To evaluate assay accuracy, any possible interference of the components within the extract with antibody binding was analyzed through the recovery of exogenous cortisol or DHEA-S added to pooled hair extracts. Each of the four reconstituted hair extracts were divided into three independent aliquots and spiked with three different known cortisol or DHEA-S concentrations, mixed, and assayed. The percentage of recovery was determined as follows: amount observed/amount expected × 100, where the amount observed is the value obtained from the spiked sample and the amount expected is the calculated amount of standard hormone added plus the amount of endogenous hormone in the unspiked sample. The recovery rates were 101.2 ± 15.7% and 114.9 ± 14.8% (mean ± SD) for cortisol and DHEA-S, respectively. The measured hormone concentrations in the spiked samples correlated with the expected concentrations: r was 0.99 for both cortisol and DHEA-S, and the model was given by the equation y = 1.00x − 1.71 and y = 0.99x + 2.04 for cortisol and DHEA-S, respectively.

To determine the parallelism between cortisol or DHEA-S standards and endogenous cortisol or DHEA-S in hair, samples containing high concentrations of endogenous cortisol or DHEA-S (100 μL) were serially diluted in 0.05 M PBS, pH 7.5, to obtain volumes of 50, 25, and 10 μL. The relationship between hair cortisol or DHEA-S concentrations, and the standard cortisol or DHEA-S curve determined through linear regression, was linear: the correlation coefficient (r) was 0.99 for both cortisol and DHEA-S, and the model was given by the equation y = 0.92x + 6.34 and y = 1.03x − 2.74 for cortisol and DHEA-S, respectively. Table 1 reports the validation parameters for the radioimmunoassay of cortisol and DHEA-S from hair.

### 2.7. Statistical Analysis

The statistical analyses were performed using R software ver. 4.0.4 (R Core Team, 2021) and SPSS software ver. 17 (SPSS Inc., Chicago, IL, USA). The normality of the data distribution was tested using the Shapiro–Wilk test. Because the distribution of the data did not follow a normal distribution, nonparametric tests were used.

In particular, differences between healthcare workers and patients were tested using the Mann–Whitney U test and Chi-squared test for continuous and categorical variables, respectively. Furthermore, considering the hair hormone concentrations and ratios, these differences were also tested using the Quades ranked analysis of covariance where the health state (healthcare workers vs. patients) and gender (male vs. female) were considered as fixed and block factors, respectively, and the age was treated as a covariate. To assess whether hormone concentrations and ratios could distinguish patients from healthcare workers on a probabilistic basis, a non-parametric receiver operating characteristic (ROC) curve analysis was performed, and the area under the curve (AUC) was calculated. The ROC curve considered sensitivity against 1-specificity, as explained by Greiner et al. [23]. The maximum Youden index [24], which maximizes the difference between true positives and false positives, was considered to choose the optimal cut-off values of hormone concentrations and ratios. The relationships of these cut-off values with the odds ratio of s were assessed using univariate binary regression. In order to consider the adjustment for the other variables (age and gender), the multivariate binary regression analysis was taken into account. In addition, the association between the number of people in the patients in relation to the cut-off of the cortisol/DHEA-S ratio was assessed using a Chi-squared test. A probability of *p* ≤ 0.05 was considered statistically significant.

## 3. Results

Table 2 reports on the characteristics of the people considered, stratified by health status. Compared to the healthcare workers, the patients were older (*p* < 0.01) and had higher cortisol concentrations (*p* < 0.01) and cortisol/DHEA-S ratios (*p* < 0.01), but lower DHEA-S concentrations (*p* < 0.05). Conversely, the proportions of males and females (*p* > 0.05) were similar between the experimental groups. Then, age and gender were included in the statistical model in order to control for their possible confounding effect on hair hormone concentrations. In this case, the patients showed higher cortisol concentrations (*p* < 0.01) and cortisol/DHEA-S ratios (*p* < 0.01) than the healthcare workers, but similar DHEA-S concentrations.

The ROC analysis revealed that the variables considered were able to distinguish between the patients and the healthcare workers (Table 3). In particular, the AUC values, which identify the probability that a randomly selected subject in the patients has a higher (cortisol and cortisol/DHEA-S ratio) or lower (DHEAS) value than a randomly selected subject among healthcare workers, were found to be 0.741 (*p* < 0.01), 0.571 (*p* < 0.05), and 0.766 (*p* < 0.01) for cortisol, DHEA-S concentrations, and cortisol/DHEA-S ratios, respectively. The cut-off values were 20.45 pg/mg, 7.65 pg/mg, and 1.46 for cortisol, DHEA-S, and the cortisol/DHEA-S ratio, respectively. However, following the suggestions of Greiner et al. [23], while the cortisol concentration and cortisol/DHEA-S ratio tests were moderately accurate, DHEA-S concentrations could less accurately distinguished between the experimental groups.

Significant associations between the patients and high cortisol concentrations (>20.45 pg/mg; *p* < 0.01), high cortisol/DHEA-S ratios (>1.46; *p* < 0.01), and low DHEA-S concentrations (<7.65 pg/mg) were found (Table 4). Conversely, gender was not associated with the patients (*p* > 0.05). When adjusted for age and gender, higher HC concentrations and cortisol/DHEA-S ratios still remained strongly and positively associated with the patients, whereas the DHEA-S concentrations showed only a negative but not statistically significant association with the patients (*p* > 0.05). Interestingly, this means that the cortisol concentration and cortisol/DHEA-S ratio were independent risk factors for the patients, but the DHEA-S concentration was not.

Considering the previously calculated cut-off value reported in Table 3, Table 5 showed the characteristics of people divided by high (>1.46) and low (<1.46) cortisol/DHEA-S ratios and stratified by health status.

Within patients, people with a high cortisol/DHEA-S ratio (>1.46) had higher cortisol concentrations (*p* < 0.01) and similar DHEA-S concentrations (*p* > 0.05) compared to healthcare workers. Conversely, patients with a low cortisol/DHEA-S ratio (<1.46) had higher cortisol concentrations (*p* < 0.01) and DHEA-S concentrations (*p* < 0.05) than healthcare workers. Similar results were found when the hormone concentrations were adjusted for age and gender.

The percentage of patients with a low cortisol/DHEA-S ratio (<1.46), (24%) was lower than that of the healthcare workers (76%; *p* < 0.01; data not reported in tables). Conversely, the number of patients (64%) with a high cortisol/DHEA-S ratio (>1.46) was higher than that of the healthcare workers (36%; *p* < 0.01; data not reported in the tables).

## 4. Discussion

To our knowledge, this is the first study in which the cortisol/DHEA-S ratio was evaluated in these populations and during the COVID-19 pandemic. Our results show that the patients had significantly higher cortisol/DHEA-S ratios than the workers. The cortisol/DHEA-S ratio in the patients reflects higher cortisol concentrations (*p* < 0.001) and lower DHEA-S (*p* < 0.05) concentrations compared to those of the healthcare workers, suggesting that they may be exposed to a greater degree of stress and a decrease in their ability to cope with their disease. Under conditions of chronic medical illness, concentrations of DHEA-S and cortisol have been observed to become dissociated [25]; cortisol secretion increased while that of DHEA-S decreased [26]. Significantly higher cortisol in patients than in healthcare workers could be linked to a non-homeostatic condition caused by the pathologies affecting the patients. Moreover, it has been described that low DHEA-S and high cortisol concentrations contribute to an increase in allostatic load [27]. Long-term alteration of the cortisol/DHEA-S ratio can have a significant impact on health, disease, and the increased risk of mental disorders [27,28,29]. Several authors have observed significantly higher cortisol concentrations in people suffering from various diseases such as multiple sclerosis [30], Alzheimer’s [31], post-traumatic stress disorder (PTSD) [32], and mental health problems [33]. Low concentrations of DHEA-S have been reported in Alzheimer’s disease [12], depression [34], and PTSD [35]. Furthermore, an increased cortisol/DHEA-S ratio was found to accelerate atherosclerosis-related diseases and to be predictive of cardiovascular diseases and all-cause mortality [36].

On the other hand, a lower cortisol/DHEA-S ratio in healthcare workers suggests their ability to maintain physiological equilibrium (homeostasis) and resilience even in the worst possible working environment for healthcare workers during the COVID-19 pandemic. Several studies have highlighted how health workers, despite the working conditions and COVID-19, have been able to maintain their mental, emotional, and physical health [37,38,39] and adopt a correct resolution of stress [40].

In our study, we evaluated the influence of factors such as health status, gender, and age on hormonal concentrations. However, it must be underlined that other factors may impact allostatic load, since it is not specific to neurological degenerative disease and represents a complex and multifactorial condition of imbalance [41].

In the population with a cut-off cortisol/DHEA-S ratio of >1.46, we found that 36% of healthcare workers lost their homeostatic condition. A high ratio is synonymous with the breaking of an optimal ratio, mainly due to the inability of DHEA-S to counteract the effects of stress that could lead to various pathologies. These workers do not perceive themselves as sick, but their condition could lead to the development of metabolic diseases [28], sarcopenia [42], high blood pressure and/or hypertension [43], neurodegenerative disease [13], and cardiovascular [44] and immune dysregulations [28]. The reduced production of DHEA-S could, therefore, be one of the links between stress and poor health [45]. In agreement with our results, two recent reports on healthy adults who experienced long-term stress have shown higher plasma cortisol/DHEA-S ratios [46,47]. In addition, Jeckel et al. (2010) [48] observed, in the caregivers of neurological patients, an increase in the salivary cortisol/DHEA-S ratios in response to chronic stress compared to non-caregivers.

Our results also reveal that 24% of the patients’ values are below the cut-off cortisol/DHEA-S ratio. These patients had higher DHEA-S concentrations than the health workers (19.95 vs. 16.80 (median; pg/mg, respectively, for the patients and health workers (*p* < 0.05)). Moreover, Ysrraelit et al. (2008) [49] observed higher concentrations of DHEA-S in patients suffering from a degenerative neurological disease than in the controls. In our opinion, despite their pathology, their difficulties, and some degree of distress, they are trying to maintain their homeostatic condition. Indeed, it has been suggested that DHEAS may act as a “buffer hormone” to preserve homeostatic balance [50]. DHEAS promotes psychological resilience and could counter the actions of cortisol as well as exert antioxidant and anti-inflammatory effects [51,52]. Given its importance, has been identified that two factors (exercise and food intake) are possible stimulators of DHEAS production [53,54,55]. Moreover, providing a supportive environment through crisis management training, providing adequate equipment and manpower, and motivating healthcare workers to achieve psychological growth during the pandemic can help them manage stress and resilience [56,57], while educating and encouraging adaptive coping strategies seems to be especially relevant in a population of chronically ill patients [58].

Compared to the majority of previous studies that use point matrices, which require repeated measurements, this is a newer, more efficient method that reflects cumulative stressor exposure up to several months prior to sampling. The concentrations of biomarkers analyzed in hair allow for a retrospective, cumulative, and objective determination of an individual’s exposure to chronic stressful events [15,59]. The sampling of a lock of hair is not an invasive or painful method, and the samples can be easily stored at room temperature for an extended period [16]. Furthermore, a hair sample can be taken alone as well as with the family or in the hospital. Assaying cortisol and DHEA-S in hair is particularly interesting because these two hormones show a circadian rhythm, and their analysis in this matrix is not affected by biorhythms.

The current study shows that the analysis of these biomarkers in hair is a useful and strategic approach to identifying a non-homeostatic and prodromic condition with the development of various pathologies both in healthcare workers and in the patients. In addition, this approach may apply to other high-stress occupations in order to address stress-related health risks and implement targeted interventions for enhancing employees’ overall health and work performance. There are a few limitations in our study. One limitation is that only a single measure of hair has been performed in our experimental design. Secondly, additional psychological and lifestyle factors (such as normal sleep, regular physical activity, and normal BMI) measures could have been instruments to capture features associated with physiological status.

## 5. Conclusions

The ability of the hair matrix to assess the cortisol/DHEA-S ratio in healthcare workers and their patients during the COVID-19 pandemic is an interesting approach. According to the results of this study, the healthcare workers had a significantly lower cortisol/DHEA-S ratio than the patients during COVID-19 pandemic. However, the cut-off allowed us to identify that 33% of the healthcare workers have lost their ability to maintain balance and resilience towards stressful events in a critical period, such as that of the pandemic. This condition can affect their quality of work and relations with their patients, but also could lead to the development of diseases.

Given that this was a case study and given the complexity and interdependence of endocrine systems, further studies are necessary. This study, however, points out the importance of the cortisol/DHEA-S ratio in hair in that it allows for identifying non-homeostatic conditions that could lead to disease, aids in the understanding of psychophysical well-being, and plays a crucial role in preventive and personalized medicine.

Hence, given the importance of identifying non-homeostatic conditions, especially in patients affected by chronic diseases or in healthcare workers, future research should increase the sample size and investigate additional biomarkers to improve the robustness of the study and comprehensively understand endocrine interactions beyond the pandemic period.

## Figures and Tables

**Table 1:** Validation parameters for the radioimmunoassay of cortisol and DHEA-S from hair.

	Cortisol	DHEA-S
Parallelism (range, equation, *r*^2^) ^a,b^	13.6–71.7 pg/well, y = 0.92x + 6.34, 0.99	6.0–70.3 pg/well, y = 1.03x − 2.74, 0.99
Recovery ^c^	101.2 ± 15.7%	114.9 ± 14.8%
Inter-day CV (*n* = 20) ^d^	13.9%	10.3%
Intra-day CV (*n* = 20) ^d^	6.4%	7.0%
Sensitivity of assay (pg/mL) ^e^	24.6	15.8

^a^ Equation of the linear regression model. ^b^ *r* was correlation coefficient. ^c^ The average concentration ($\bar{x}$ ± SD) is shown. Percentage recovery was calculated on observed vs. expected. ^d^ Intra-day and inter-day precision was estimated using coefficient of variation (CV). ^e^ Determined as the hormone concentration that resulted in the displacement of the labeled hormone by at least two SD from maximal binding (as calculated by RiaSmart; Canberra-Packard).

**Table 2:** Characteristics of people stratified by health.

	Total	Health (H)		*p*-Values	*p*-Values
		Healthcare Workers *n* = 200	Patients *n* = 161		
Age, y	63 [50–78]	55 [40–64]	77 [65–83]	<0.001 *	-
Female, *n*	250	137	113	0.819 ***	-
Male, *n*	111	63	48		
Cortisol, pg/mg	19.90 [8.80–34.70]	12.35 [7.65–24.00]	27.40 [18.00–48.30]	<0.001 *	<0.001 **
DHEA-S, pg/mg	12.30 [6.30–20.60]	13.2 [7.58–24.71]	10.70 [5.80–18.70]	0.021 *	0.981 **
Cortisol/DHEA-S ratio	1.52 [0.79–2.84]	1.02 [0.60–1.76]	2.53 [1.41–5.50]	<0.001 *	<0.001 **

* Differences tested between healthcare workers and patients using Mann–Whitney U test; ** Differences tested between healthcare workers and patients including age and gender as covariate and block factor, respectively, in Quades ranked analysis of covariance; *** associations between gender and health was assessed with Chisquare test. Data were expressed as medians [quartile 25–75% value] or numbers.

**Table 3:** Receiver operating characteristic (ROC) curve analysis with determination of area under the curve (AUC) and cut-off values of hair hormone concentrations and ratio.

	Cut-Off Values	AUC (95% CI)	*p* Values
Cortisol, pg/mg	20.45	0.741 (0.690–0.792)	<0.001
DHEA-S, pg/mg	7.65	0.571 (0.512–0.630)	0.021
Cortisol/DHEA-S ratio	1.46	0.766 (0.716–0.817)	<0.001

**Table 4:** Odds ratio (OR) for patients.

	Before Adjustment *		After Adjustment **	
	OR (95% CI)	*p*-Values	OR (95% CI)	*p*-Values
Gender, Female	1.08 (0.69–1.70)	0.730	-	-
Cortisol > 20.45, pg/mg	5.24 (3.34–8.23)	<0.001	7.11 (3.96–12.76)	<0.001
DHEA-S < 7.65, pg/mg	1.83 (1.17–2.87)	0.008	1.57 (0.91–2.72)	0.104
Cortisol/DHEA-S ratio > 1.46	5.75 (3.63–9.10)	<0.001	5.20 (2.96–9.13)	<0.001

* Binary regression analysis; ** regression analysis adjusted for age and gender.

**Table 5:** Characteristics of people with hair cortisol/DHEA-S ratio > 1.46 or <1.46 stratified by health.

	Total	Health (H)		*p*-Values *	*p*-Values **
		Healthcare Workers	Patients		
Cortisol/DHEA-S > 1.46					
Cortisol, pg/mg	28.30 [19.05; 48.20]	22.75 [9.68; 36.10]	34.50 [21.60; 60.10]	<0.001	0.002
DHEA-S, pg/mg	8.30 [5.05; 14.80]	9.65 [4.98; 15.04]	7.50 [5.10; 14.70]	0.625	0.462
Cortisol/DHEA-S < 1.46					
Cortisol, pg/mg	10.20 [7.13; 20.58]	9.90 [6.80; 16.83]	19.15 [8.10; 24.30]	0.006	0.004
DHEA-S, pg/mg	17.95 [10.03; 30.08]	16.80 [8.50; 27.75]	19.95 [13.98; 33.93]	0.048	0.005

* Differences tested between healthcare workers and patients using Mann–Whitney U test; ** Differences tested between healthcare workers and patients including age and gender as covariate and block factor, respectively, in Quades ranked analysis of covariance. Data were expressed as medians [quartile 25–75% value].

## Data Availability

The data presented in this study are available on request from the corresponding author (for privacy, legal, or ethical reasons).
